# *OeFAD8, OeLIP* and *OeOSM* expression and activity in cold-acclimation of *Olea europaea*, a perennial dicot without winter-dormancy

**DOI:** 10.1007/s00425-016-2490-x

**Published:** 2016-02-26

**Authors:** Simone D’Angeli, Maya Matteucci, Laura Fattorini, Angelo Gismondi, Matteo Ludovici, Antonella Canini, Maria Maddalena Altamura

**Affiliations:** Dipartimento di Biologia Ambientale, Università ‘Sapienza’, P.le A. Moro 5, 00185 Rome, Italy; Dipartimento di Biologia, Università degli Studi di Roma “Tor Vergata”, Via della Ricerca Scientifica 1, 00133 Rome, Italy

**Keywords:** Cuticle, Fatty acid desaturase (FAD8), Linolenic acid, LIP transcription factor, Lipidome, Osmotin

## Abstract

**Cold-acclimation genes in woody dicots without winter-dormancy, e.g., olive-tree, need investigation. Positive relationships between*OeFAD8***, ***OeOSM*, and*OeLIP19*and olive-tree cold-acclimation exist, and couple with increased lipid unsaturation and cutinisation.**

Olive-tree is a woody species with no winter-dormancy and low frost-tolerance. However, cold-tolerant genotypes were empirically selected, highlighting that cold-acclimation might be acquired. Proteins needed for olive-tree cold-acclimation are unknown, even if roles for osmotin (OeOSM) as leaf cryoprotectant, and seed lipid-transfer protein for endosperm cutinisation under cold, were demonstrated. In other species, *FAD8,* coding a desaturase producing α-linolenic acid, is activated by temperature-lowering, concomitantly with *bZIP*-*LIP19* genes. The research was focussed on finding *OeLIP19* gene(s) in olive-tree genome, and analyze it/their expression, and that of *OeFAD8* and *OeOSM,* in drupes and leaves under different cold-conditions/developmental stages/genotypes, in comparison with changes in unsaturated lipids and cell wall cutinisation. Cold-induced cytosolic calcium transients always occurred in leaves/drupes of some genotypes, e.g., Moraiolo, but ceased in others, e.g., Canino, at specific drupe stages/cold-treatments, suggesting cold-acclimation acquisition only in the latter genotypes. Canino and Moraiolo were selected for further analyses. Cold-acclimation in Canino was confirmed by an electrolyte leakage from leaf/drupe membranes highly reduced in comparison with Moraiolo. Strong increases in fruit-epicarp/leaf-epidermis cutinisation characterized cold-acclimated Canino, and positively coupled with *OeOSM* expression, and immunolocalization of the coded protein. *OeFAD8* expression increased with cold-acclimation, as the production of α-linolenic acid, and related compounds. An *OeLIP19* gene was isolated. Its levels changed with a trend similar to *OeFAD8.* All together, results sustain a positive relationship between *OeFAD8, OeOSM* and *OeLIP19* expression in olive-tree cold-acclimation. The parallel changes in unsaturated lipids and cutinisation concur to suggest orchestrated roles of the coded proteins in the process.

**Electronic supplementary material:**

The online version of this article (doi:10.1007/s00425-016-2490-x) contains supplementary material, which is available to authorized users.

## Introduction

Some plants are able to tolerate near zero/subzero temperatures by the process known cold-acclimation, acquired by exposure to progressively lower, non-freezing, temperatures up to the acquisition of a freezing tolerance (Browse and Xin 2001). Acclimation acquisition is genotype-dependent (Sun et al. 2009). How plants sense low temperatures and decode this message into transcriptome changes leading to acclimation still remains a widely unresolved question (Knight and Knight 2012). A lot of genes have been found to be active in cold-acclimation of herbaceous plants, e.g. *Arabidopsis* and winter cereals (Chinnusamy et al. 2010, and references therein), whereas knowledge about genes active in woody plants is much more limited. Moreover, in woody perennials, cold acclimation is generally coupled with endo-dormancy acquisition, and deciduousness, with these processes, possibly sharing gene activity, and together causing tree survival in winter under prolonged subzero temperatures (Welling and Palva 2006). However, there are also trees, which are evergreen, lack winter-dormancy, but remain able to cold acclimate (Arora et al. 1992). The molecular induction/regulation of cold-acclimation in such perennial plants is widely unknown, and particularly in fruiting dicots. Olive tree is a woody dicot of the Mediterranean Basin without winter-dormancy, and with economic value for the oil of its drupe. The areas of the Mediterranean Basin account for more than 90 % of the world olive oil production (Hatzopoulos et al. 2002), however cultivation is progressively extending to countries uncharacterized by the Mediterranean climate, where the trees have to survive to colder winters (Matteucci et al. 2011, and references therein). Olive tree is a cultigen complex (Green 2002), composed of genotypes retaining genetic characteristics over hundreds/thousands of years, because a vegetative propagation applied over centuries (Hatzopoulos et al. 2002). It is a species maintaining the leaves all over the year, and the drupes for months during fall/early winter. Olive tree exhibits a general low cold-tolerance with serious problems for survival below −12 °C (D’Angeli et al. 2003, and references therein). However, in colder regions of its distribution area, there are genotypes, empirically selected, which produce a better oil because an increased formation of ω3 and ω6 unsaturated fatty acids (FAs) in the drupe (Matteucci et al. 2011). Leccino, Canino and Picual are among the genotypes traditionally known as cold-tolerant, Frantoio has been reported to be semi-tolerant, and Moraiolo and Taggiasca cold-sensitive (D’Angeli et al. 2003; de la O Leyva-Pérez et al. 2015), however, the genetic base of their cold-resistance differences are still widely unknown.

Transient increases in cytosolic calcium ([Ca^2+^]_cyt_) are among the early responses to low temperatures, ceasing when cold acclimation is acquired (Knight and Knight 2012). In accordance, exogenously applied calcium chelators inhibit acclimation, whereas calcium ionophores induce it at higher temperatures, as in alfalfa (Monroy and Dhindsa 1995). Because [Ca^2+^]_cyt_ transients mainly occur by apoplastic calcium influx, the protoplasts are more suitable than the whole cells for their monitoring, being devoid of calcium rich cell walls, and similarly viable (Monroy and Dhindsa 1995, and references therein; D’Angeli et al. 2003, and references therein). Moreover, the cold-shock-induced [Ca^2+^]_cyt_ response by protoplasts is representative of the whole plant, as shown in *Nicotiana plumbaginifolia* by comparing leaf protoplasts with plants constitutively expressing the recombinant calcium indicator aequorin which produces a luminescence directly related to [Ca^2+^]_cyt_ (Mazars et al. 1997). Cold-induced changes in [Ca^2+^]_cyt_ have been also found in olive tree leaf and drupe protoplasts from numerous genotypes (D’Angeli et al. 2003; D’Angeli and Altamura 2007; Matteucci et al. 2011).

The cuticle provides a protective hydrophobic coating for aerial plant organs and plays a pivotal role during plant interaction with the environment, with dynamic qualitative and quantitative changes (Borisjuk et al. 2014; Martin and Rose 2014). Water deficit has been reported to trigger an increase in cuticular thickness as an adaptation response to summer drought, e.g. in numerous species of the Mediterranean region, including olive tree (Bacelar et al. 2004). Moreover, in many broad-leaf evergreens, the formation of a thick cuticle also occurs as protection to the cold-induced winter drought (Goodwin and Jenks 2005). Cutin, its major component, prevalently consists of hydroxyl-FAs, derived by C16- and C18-FAs and glycerol-molecules (Borisjuk et al. 2014). Fruit and leaf cuticles generally contain the same classes of compounds, however, genotype-specific changes may occur during the stress responses, e.g., in olive tree genotypes in response to drought and pathogens (Bacelar et al. 2004; Gomes et al. 2012). By [Ca^2+^]_cyt_-signalling investigations in cold-shocked protoplasts, and overexpression/immuno-localization studies, the tobacco PR-5 protein osmotin was proposed as cryoprotectant for olive-tree leaves because its over-expression blocked cold-induced [Ca^2+^]_cyt_-transients in non-cold-acclimated protoplasts (D’Angeli and Altamura 2007). An osmotin-like protein with cryoprotective activity has been also found in *Solanum dulcamara* leaves, and a role in freezing tolerance has been suggested (Newton and Duman 2000), although the mechanism of action not yet determined. An osmotin, associated with cuticle biogenesis, is active in tomato fruit (Yeats et al. 2010), and an olive tree osmotin (OeOSM) has been found to be active in olive tree seed (D’Angeli et al. 2013). In the latter, oleic (C18:1) and linoleic (C18:2) FAs, produced by specific FA-desaturases (FADs), are components of the endosperm-cuticle, which increases under temperature-lowering, and OeOSM is involved in the lipid-trafficking necessary for this thickening (D’Angeli et al. 2013). Taken together, there is the possibility that OeOSM is involved in the cold-acclimation of olive tree leaves/drupes through a role on cutinisation, possibly also requiring specific FAD(s) activity(ies).

FAD-activity is also required for the membrane restructuring related to cold-acclimation, because increased desaturation of glycerolipids serves as compensation to cold-caused decrease in membrane fluidity (Szymanski et al. 2014). In olive tree, numerous *OeFAD* genes are expressed in the drupe during the oil production phase, ceasing in expression at the end of this phase, and, among them, *OeFAD2.2*, and *OeFAD7* show an increased expression in response to cold (Matteucci et al. 2011), but are not related with cold acclimation. In fact, these genes, coding FADs for the conversion of C18:1 into C18:2, and C18:2 into C18:3 (α-linolenic acid), respectively, are active in leaves of the cold-tolerant Picual during early cold-exposure, but down-regulated under long lasting cold-exposure (de la O Leyva-Pérez et al. 2015). The same genes are up-regulated in cold-stressed drupes of both Canino and Moraiolo, but always transiently (Matteucci et al. 2011). In *Arabidopsis* two *ω3*-*FAD* genes, i.e., *AtFAD7* and *AtFAD8,* encode two *ω3*-FAD plastidial isozymes which are the major contributors of C18:3 in the leaves (McConn et al. 1994). Despite a high degree of structural relatedness, the activities of the two isozymes are regulated differentially by temperature, with AtFAD8 induced by the low temperatures (Matsuda et al. 2005 and references therein). Increases in 18:3/18:2 ratio in leaves/fruits have been associated with an enhanced cold-tolerance also in tomato (Domínguez et al. 2010), and in other plants, e.g. rice, *FAD8* transcript levels rise under low temperatures (Wang et al. 2006). An *OeFAD8* gene has been found in olive tree (Poghosyan et al. 1999; Matteucci et al. 2011), but it is expressed only in traces during the oleogenic phase of the drupe (Matteucci et al. 2011). However, this does not exclude that it may be specifically active during olive tree cold-acclimation.

Low-temperature-induction is a feature of the *LIP19* (*L*ow-*T*emperature *I*nduced *P*rotein) subfamily members of the basic–domain leucine zipper (bZIP) transcription factors (TFs; Yang et al. 2001). A positive relationship between cold-induced [Ca^2+^]_cyt_-signalling and the expression of genes of the subfamily has been demonstrated (Berberich and Kusano 1997; Ito et al. 1999). In wheat, the expression of a *LIP19* is higher in freezing-tolerant genotypes than in cold-sensitive ones, and the gene over-expression improves freezing-tolerance in tobacco (Kobayashi et al. 2008). Moreover, the expression under low temperatures of a *LIP19* in maize increases concomitantly to that of *ZmFAD8* (Berberich et al. 1998), suggesting a possible relationship between the two genes. No of *LIP19* TFs has yet been identified in olive tree.

The present research aimed to find *LIP19* gene(s) in olive-tree genome, and analyze it/their expression, and those of *OeFAD8* and *OeOSM,* under different cold-conditions/drupe-developmental stages, in comparison with possible changes in unsaturated lipids and cell wall cutinisation, in drupes and leaves of genotypes traditionally known for a different cold-tolerance, with the goal of establishing whether and how these genes were involved in cold-acclimation.

Results showed that an *OeLIP19* gene was present in olive tree genome, and that, together with *OeFAD8* and *OeOSM,* was involved in cold-acclimation starting from its induction. Changes in unsaturated lipids and cutinisation paralleled the changes in the transcription of these genes, suggesting orchestrated roles of the coded proteins in the cold acclimation process.

## Materials and methods

### Plant growth conditions

Trees of *Olea europaea* L. cv. Canino, endemic of the volcanic areas of Northern Lazio, cv. Leccino from Northern Tuscany, cv. Frantoio from Southern Tuscany, cv. Moraiolo from Central Italy, and cv. Taggiasca from Ligury (Jacoboni and Fontanazza 1981) were grown in pots under the same conditions (i.e., standard sandy loam soil, daily irrigation) in the Botanical Garden of the Environmental Biology Department (Sapienza University, Rome, 41°53′33.5″ latitude North, 12°29′31″ longitude West, 20 meters above sea level). The trend of mean temperature-changing was similar in the years of the experiment (http://www.arsial.it/portalearsial/agrometeo). The experiments were planned in the way that each year 6-year-old specimens were used per genotype. Each year, the genotypes synchronously showed full blooming (last week of May), and drupe development during the weeks after flowering (WAFs).

In the first 2 years (2009–2010), specimens from all genotypes were used, whereas in the lasting 3 years (2011–2013) only those from cv. Canino, cv. Moraiolo and cv. Frantoio.

### Cold treatments applied to the plant

Three trees per experiment coming from the open-air stocks of Canino and Moraiolo were put at specific WAFs in a plant growth-chamber XL15E (Labco, Milan, Italy), under 16 h/8 h (light/dark) photoperiod (300 μmol photons m^−2^ s^−1^), and exposed to cold-treatments of various intensity/duration. In detail, plants at the beginning of oleogenesis in the drupes, i.e. at WAF 10 (Matteucci et al. 2011), when the open-air temperature was 25 °C, were put in the growth-chamber at 6 °C either for 6 h (A_1_-type cold treatment), or for 24 h (A_2_-type cold treatment), or for 72 h (A_3_-type cold treatment). Alternatively, the plants were exposed at 0 °C for 2 h, and then at 10 °C for 14 days (B-type cold treatment). The controls were kept at 25 °C. In some cases A_3_-type cold treatment was also applied at WAF19 (end of oleogenesis in the drupes, Matteucci et al. 2011).

Full winter plants (WAF26) were also put in the growth-chamber, and progressively exposed to a temperature decrease from 7 °C (open-air temperature) to −10 °C into 35 h, maintained at −10 °C for 2 h, and then exposed to a temperature increase up to 7 °C into other 35 h (C-type cold treatment). The controls were kept at 7 °C. WAF 26 was chosen because it was several weeks after the end of oleogenesis (Matteucci et al. 2011).

### Protoplast isolation, viability, and fluorescence measurements

Protoplasts were obtained from leaves from the fourth apical node of the youngest twigs [i.e., those that had been elongated in the spring of the year of the experiment (twigs of the year according to D’Angeli et al. 2003)], and/or from drupes (epi-mesocarp), collected at specific WAFs, i.e. WAF10 (beginning of oleogenesis), WAF19 (end of oleogenesis), WAF22 (drupe maturation, Matteucci et al. 2011), and WAF26 (full winter). Protoplast isolation was performed at 22 °C room temperature both for specimens coming from open-air and for those exposed to cold-treatments of various intensity/duration. Cell viability after isolation was evaluated with fluorescein diacetate (values higher than 70 % in each genotype). The protoplasts were incubated in the same enzymatic solution of isolation. The variations in [Ca^2+^]_cyt_ after cold-shocks of cooling rate (Δ*T*/Δ*t*) (Plieth et al. 1999) of 10 °C/60 s starting from 22 °C were detected incubating the protoplasts with Calcium-Crimson-AM fluorochrome (5 μM, Invitrogen, Italy). The fluorescence signal was evaluated by means of a Zeiss Axiolab (Carl Zeiss, Italy) epifluorescent microscope equipped with a DFC350F (Leica Microsystems, Italy) cooled camera, quantified with an Optilab 2.6 software (Graftek, Mirmande, France), and expressed as average pixel intensity (D’Angeli et al. 2003). Two technical replicates per year and genotype were carried out (data from the first replicate of the second year shown).

### Electrolyte leakage procedure

Electrolyte leakage test is a means to assess the extent of plant injury in relation to low temperature exposure. Electrolyte diffusion from cells increases when membranes are damaged, and is reduced in the presence of cold acclimation (Lindén et al. 2000; Rohde et al. 2004). The samples are frozen and then thawed, and the electrolyte (ion) leakage in the bathing solution measured. The freeze–thaw damage is quantified as the mean percentage of the conductivity of the bathing solution relative to the conductivity of the solution after releasing all electrolytes from membranes by boiling (Rohde et al. 2004, and other references therein). Briefly, three WAF10-plants per olive tree genotype (Canino and Moraiolo) were exposed to either A_3_-type or B-type cold treatment, and other three plants maintained under the open-air temperature of the WAF (control plants). Other three WAF19-plants per genotype, coming from the open air stocks, were used for the test. Discs (5-mm-in-diameter) were excised from the leaf lamina and the drupe epi-mesocarp, and one gram/fresh weight per organ type was frozen at a rate of 2 °C/h up to −10 °C, and maintained at −10 °C for 30 min (Rohde et al. 2004). The frozen samples were then slowly thawed on ice. Parallel samples were kept on ice as unfrozen controls. All samples were then immersed in distilled water and placed on a shaker for either 24 or 16 h (Rohde et al. 2004) at 4 °C under darkness. Electrolyte leakage was determined at room temperature (22 °C) using an HI 8333 conductivity meter (Hanna Instruments), and expressed as mean percentage (±SE) of conductivity of the bathing solution before boiling relative to the after-boiling conductivity of the same solution. Because no difference in ion leakage percentage was present between shaking at 24 and 16 h, the three technical replicates for experiment here reported were carried out with a 16 h shaking.

### Lipid detection, histological staining, and OeOSM immunolocalization

Five samples of epi-mesocarp and leaves per genotype (Canino and Moraiolo) were selected randomly at WAF19, and 50 mg/dry weight (DW) of each sample extracted. The samples were split in two aliquots. One aliquot was used for quantitative analysis of oxylipins by LC–MS/MS, as reported by Ludovici et al. (2014), and the other one for the quantification of α-linolenic acid [C18:3 (n-3)] as free FA (FFA), and FA bound in triacylglycerol (TAG) and polar lipid (PL) fractions by GC-FID analysis, according to D’Angeli et al. (2013) (data from two independent determinations per year and genotype, results of the third year shown).

Ten drupes and leaves per year and genotype (Canino, Moraiolo, and Frantoio) were randomly collected at WAF19 and WAF22, hand-cut, and oil bodies and cutin detected by SUDAN IV staining. Epi-mesocarp and leaf samples were either fixed, dehydrated, embedded, sectioned, and stained with toluidine blue, or attached on a scanning electron microscopy stub, and treated as in Matteucci et al. (2011). Moreover, OeOSM immuno-localization was carried out in resin embedded leaf and drupe samples according to D’Angeli and Altamura (2007), and the absence of staining verified in the controls treated without the primary antibodies (data not shown). Images were acquired as in Matteucci et al. (2011). Two technical replicates per year were carried out, data from the first one of the third year shown.

### RNA extraction, cDNA synthesis, real time PCR (qPCR), gene sequencing and alignment

Total RNA was isolated from ten drupes (epi-mesocarp) and leaves of Canino and Moraiolo, collected from specimens either grown under open-air, or exposed to A- to C-types of cold-treatments, as specified in the Results. Moreover, at WAF26 ten leaves per genotype were excised from open-air grown plants and incubated in 5 μM solution of A23187 calcium ionophore (Sigma; D’Angeli et al. 2003) for 72 h before RNA extraction.

The cDNA was synthesized and applied in qPCR analysis through a Line Gene 9620 (Bioer, Binjiang, China), according to Matteucci et al. (2011) and D’Angeli et al. (2013). cDNAs were also used as templates for PCR amplification and sequencing of *OeLIP* and *OeFAD8* genes according to Gismondi et al. (2012, 2013). *OeLIP* nucleotide sequence was translated in amino acidic succession by ExPASy Bioinformatics Resource Portal (http://web.expasy.org/translate/). For the construction of the phylogenetic tree, various accessions of plant b-ZIP proteins (including LIPs) were obtained from the GenBank database (http://www.ncbi.nlm.nih.gov/nuccore/). The multiple sequence alignment was performed by ClustalW2.1 program (http://www.ebi.ac.uk/Tools/msa/clustalw2/) and the relative similarity matrix visualized, according to Neighbor–Joining method (Saitou and Nei 1987), by TreeView32 software (Roderic DM Page, University of Glasgow, Scotland, UK) as a phylogenetic radial tree. The primers used in qPCR and sequencing analysis were designed as described in Matteucci et al. (2011) and D’Angeli et al. (2013) starting from http://140.164.45.140/oleaestdb/index.php database (Table 1). The primer sequences for PCR amplification of *OeFAD7*, *OeFAD3*, and *OeOSM* (Matteucci et al. 2011; D’Angeli et al. 2013) were also reported in Table 1.

**Table 1:** Primer sequences used for qPCR and sequencing (OeLIP) analyses in leaves and drupes of Canino and Moraiolo

Gene	Forward primer sequence	Reverse primer sequence
OeFAD7 (HQ889832)	CCCATGTCATACATCACCTC	GCAAATACAATGGAAGAGGC
OeLIP (KR360744)	CAGCAAGCGGTGAGTTCTG	ATCCGTGACCTACGAGCA
FAD3 (HQ DQ788673)	GTCATGGGGATGTATTACAGAG	CAAAGTGGTCTTCTTTAACGC
OeOSM (E8NTSAO03G6K29)	AACACCTTGGCT GAATACGC	CGCCGTTTATAGCCGTACAT
CRY2 (E8NTSAO03GWZ77)	GTCCTACAAGCTCGTCCTATG	CTTGTCGCAACTATGCAAGT

*Cry2* used as housekeeping gene

*Cry2* was used as housekeeping gene for qPCR based on the stability of its mRNA expression in different plant tissues and developmental stages, and in presence of temperature variations, in accordance with previously reported housekeeping gene selection in the same species (Table S2 in D’Angeli et al. 2013).

Three technical replicates for two independent experiments/year were carried out for all the analyses and expressed as means (±SE). Data from the third year shown.

### Statistical analysis

A normality test was applied before analysis of variance (Instat 3, GraphPad, La Jolla, CA, USA), and one-way/two-way ANOVA (*P* < 0.05), followed by Tukey’s post test (Prism 6.0, GraphPad), used to compare mean values (±SE) of the same organ, either within the same genotype or between different genotypes. Alternatively, means (±SE) in pairs were compared by Student’s *t* test. Results were similar in all the crop years.

## Results

### Cold-shock-induced cytosolic calcium response changes in parallel in leaves and drupes, and no change in cytosolic calcium, but a reduction in ion leakage from cell membranes, occur in both organs when cold acclimation is reached

Cold-responsiveness was verified in leaves and drupes of open-air grown specimens of Canino, Leccino, Moraiolo, Frantoio, and Taggiasca genotypes at WAF10 and WAF19, i.e., at the beginning and end, respectively, of the oleogenesis, and related FAD activities, in the drupes (Matteucci et al. 2011), by monitoring [Ca^2+^]_cyt_-transients in their protoplasts under Δ*T/*Δ*t* = 10 °C/60 s cold-shocks (D’Angeli et al. 2003, and “Materials and methods”).

Transient [Ca^2+^]_cyt_-rises were always present at WAF10, but remained present only in Frantoio, Taggiasca and Moraiolo at WAF19, showing that Canino and Leccino had acquired acclimation during this WAF-interval, whereas the other genotypes had remained cold-sensitive, as exemplified by Canino and Moraiolo in Fig. S1a–d. At WAF22, also leaves and drupes of Frantoio did not show any [Ca^2+^]_cyt_-rise after the same cold-shock (Fig. S1e), showing acclimation acquisition during the interval between WAF19 and WAF22, in accordance with previous results for the drupes (D’Angeli et al. 2013).

Based on these results, the following analyses were focussed on Canino and Moraiolo because representative of the cold responsiveness of the other genotypes, after having confirmed the acquisition (Canino), and the non-acquisition (Moraiolo) of cold acclimation at WAF19 by the application of the ion leakage test (see “Materials and methods”). In fact, the mean percentage of ion leakage was 68 % (±1) in the leaves, and 79 % (±0.1) in the drupes of Canino, and 86 % (±0.1) in the leaves, and 91 % (±0.2) in the drupes of Moraiolo. The statistical analysis of data showed that the leakage percentage from the leaves was significantly (*P* < 0.0001) lower than from the drupes in both genotypes, however it was significantly (*P* < 0.0001) reduced in Canino in comparison with Moraiolo independently of the organ.

**Fig. 1:**
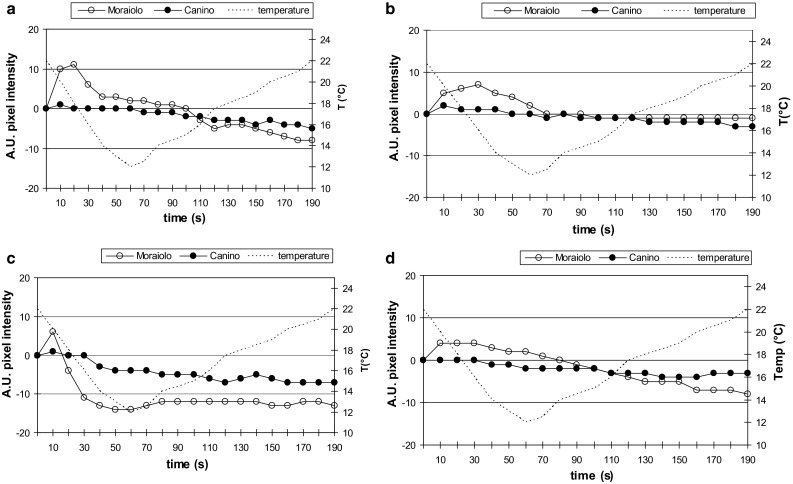
Variations in [Ca^2+^]_cyt_ expressed as pixel intensities (A.U., arbitrary units), in Canino (*solid symbols*) and Moraiolo (*empty symbols*) protoplasts of epi-mesocarp (**a**, **c**) and leaf (**b**, **d**) incubated with calcium-crimson-AM under a cold-shock of Δ*T/*Δ*t* = 10 °C/60 s from WAF10-plants that had been exposed to either an A_3_-type cold treatment (**a**, **b**) or a B-type one (**c**), and from WAF26-plants that had been exposed to a C-type cold treatment (**d**). *n* = 80. Protoplasts representative of the average response shown [6.2 (±0.3) Moraiolo mean rise in **c**]. A_3_-, B-, and C-type plant cold treatments described in “Materials and methods”

At WAF10, cold-shocks on protoplasts isolated from plants exposed to A_1_- and A_2_-types of cold treatment resulted into the persistence of [Ca^2+^]_cyt_-rises, i.e., presence of cold-responsiveness, in both genotypes (Fig. S2a, b). An A_3_-type cold treatment to the plant was still not-sufficient to cause cold-acclimation in Moraiolo, but sufficient in Canino, because, under the cold-shock, only the drupe and leaf protoplasts of Moraiolo continued to show calcium rises [10.1 (±0.8) and 8.6 (±0.8) mean values, respectively] (Fig. 1a, b). The result was confirmed by the electrolyte leakage test, because the leaves and drupes of Canino showed a mean percentage of ion leakage highly (*P* < 0.0001) reduced in comparison with Moraiolo under the same A_3_-type cold treatment to the plant (Fig. S3). Also when the plants were exposed to the B-type cold treatment before protoplast cold-shocking, Moraiolo remained cold-sensitive, differently from Canino, because only the cold-shocked protoplasts of the former showed the [Ca^2+^]_cyt_-rises (Fig. 1c). The ion diffusion from the membranes of leaves and drupes coupled with this result, because the mean percentage of ion leakage in both organs was significantly (*P* < 0.0001) higher in Moraiolo than in Canino (Fig. S3). Collectively, results from cytosolic calcium response in the protoplasts, and ion leakage from cell membranes after the B-type cold treatment to WAF10-plants, demonstrate that the sudden exposure of Canino plants to 0 °C for 2 h, coming from an open-air temperature of 25 °C, followed by a long exposure to a higher temperature, which however was fifteen degrees lower than the open-air temperature, is sufficient to induce and maintain a sudden cold acclimation in the genotype. In accordance, perennial plants without winter dormancy, as olive tree, are known to acclimate rapidly and maintain cold hardiness even under periods of unseasonably higher temperatures (Guy 1990).

To verify whether Moraiolo might become cold-acclimated later than WAF19, the cytosolic-calcium response induced by the cold-shock in the leaf protoplasts (the drupes were fallen from the tree) was evaluated at WAF26 (full winter) in the open-air grown specimens. Calcium rises continued to be present, sustaining that Moraiolo had remained cold-sensitive (Fig. S2c). When, at WAF26, the plants were exposed to a C-type cold treatment before protoplast isolation, the inability of Moraiolo to acquire cold-acclimation was confirmed, because its protoplasts still showed presence of [Ca^2+^]_cyt_-rises under cold-shocking [4.1 (±0.5) mean value, Fig. 1d)].

### Cold-acclimation couples with increased epicarp/leaf cutinisation, and OeOSM expression/immunolocalization

**Fig. 2:**
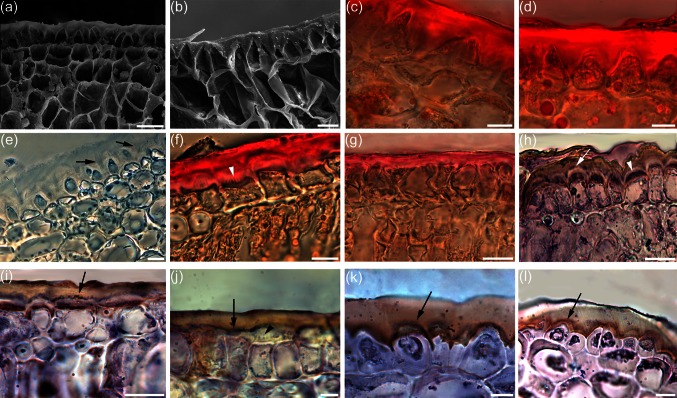
Cutinization in the outer-cell-wall of epicarp (**a**–**e**) and adaxial leaf epidermis (**f**, **g**), and OeOSM immunolocalization in the same tissues (**h**–**l**), of Canino and Moraiolo. **a**, **b** Epi-mesocarp of Moraiolo drupes at WAF3 (**a**) and WAF10 (**b**) (scanning electron microscopy). **c**, **d** Cutinized outer cell wall, and OBs in the epicarp and mesocarp cells at WAF19 after SUDAN IV staining, in Canino (**c**) and Moraiolo (**d**). **e** Detail of Canino drupe at WAF19 after toluidine blue staining (phase-contrast microscopy) showing micro-channel-like striations in the cutinized outer-cell-wall (*arrows*). **f**, **g** Details of Canino (**f**) and Moraiolo (**g**) leaf epidermis, and outer palisade, at WAF19. SUDAN IV marks the cutinization in the outer cell wall, the micro-channel-like striations in its inner part (*arrowhead*, **f**), and OBs within the cells. **h**–**l** OeOSM presence at WAF19 in the leaf epidermis and outer palisade (**h**–**j**), and in epicarp and adjacent mesocarp (**k**–**l**) of Canino (**h**, **i**, **k**), and Moraiolo (**j**, **l**). (OeOSM in the cutinized cell wall shown by the *arrows*, and in the micro-channel-like systems by the *arrowheads*). Cross-sections, *bars* 20 μm

In both Canino and Moraiolo, the epicarp outer cell wall was thickened, and with anticlinal pegs, already at WAF3 (Fig. 2a), and thickening continued during the following WAFs (Fig. 2b). Thickness became significantly higher in Canino than Moraiolo at WAF19 (Table 2), i.e., when Canino drupes had become cold-acclimated under open-air, but not those of Moraiolo (Fig. S1d). In Canino, in particular, cutinized pegs were highly extended (Fig. 2c), and the cell walls crossed by micro-channel-like striations (Fig. 2e, arrows). Only in Canino drupes, thickening continued (20 % mean increase at WAF22 in comparison with WAF19). In both genotypes, oil bodies (OBs) were present in the epicarp and adjacent mesocarp cells (Fig. 2c, d).

**Table 2:** Thickness (mean value in μm ± SE) at WAF19 of the cutinized outer cell wall of the epicarp and the adaxial leaf epidermis from the cold-sensitive Moraiolo and the cold-acclimated Canino

	Cv. Moraiolo	Cv. Canino
Drupe epicarp	22.95 ± 0.76	30.1 ± 0.67***
Adaxial leaf epidermis	9.28 ± 0.3	20.66 ± 0.9***

*** *P* < 0.0001 in comparison with the other value in the same row. *n* = 100

As for drupes, at WAF19, cutinisation of the outer cell walls of adaxial leaf epidermis was significantly higher in Canino than Moraiolo (Table 2). All together results showed that even if a thicked cuticle as an adaptation response to summer drought in numerous species of the Mediterranean region, including olive tree (Bacelar et al. 2004), there was a further increase in both the aerial organs related to cold acclimation acquisition. However, thickening in the outer cell walls of adaxial leaf epidermis did not continue at WAF22 in any genotype.

The OBs were present in the cytoplasm of both epidermal and adjacent palisade cells (Fig. 2f, g), and micro-channel-like striations appeared in the inner cutinized outer cell wall of Canino, in particular (Fig. 2f). To exclude that the increase in thickening of the outer cell wall of Canino leaf/drupe was due to peculiarities of the genotype unrelated with the acquired cold-acclimation, the thickness of the outer cell wall was also measured in Frantoio, in the absence (WAF19) and presence (WAF22) of acclimation (Fig. S1e). Results confirmed that the increased cutinisation was related to acclimation independently of the genotype, but also showed that the thickness reached at acclimation was genotype-dependent. In fact, in comparison with Canino and Moraiolo at WAF19 (Table 2), in Frantoio, at the same WAF, the outer cell walls of the epicarps and adaxial leaf epidermides showed a thickness comparable to that of the non-acclimated Moraiolo (i.e., 20.5 ± 0.9 and 9.2 ± 0.2 μm, in the two organs, respectively), whereas at WAF22 values significantly (*P* < 0.0001) higher (i.e., 27 ± 0.2 and 16 ± 0.6 μm, in the two organs, respectively), even if statistically lower (*P* < 0.0001) than those of the WAF19-acclimated Canino.

Moreover, in Canino and Moraiolo, at WAF19, OeOSM was present within the cells of the leaf adaxial epidermis and upper palisade (Fig. 2h–j), and in the epi-mesocarp (Fig. 2k–l). The immuno-localization signal was also present in the inner cutinized outer cell wall (Fig. 2h–l, arrows), including the micro-channel-like striations, and with higher intensity in Canino than Moraiolo (Fig. 2h, j, arrowheads).

**Fig. 3:**
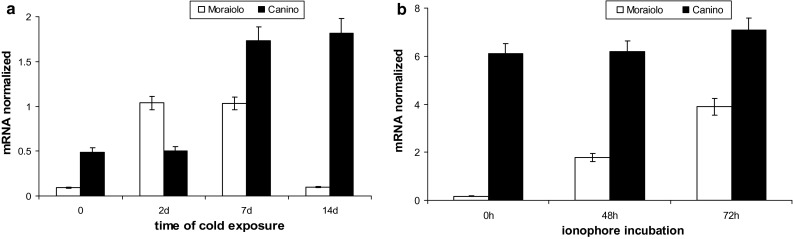
Expression profiles by qPCR of *OeOSM* transcripts in drupes of Canino (*black columns*) and Moraiolo (*white columns*) during a B-type cold treatment to WAF10-plants (**a**), and in leaves of WAF26-plants during 72 h of Ca^2+^-ionophore A23187 leaf-treatment (**b**). Data reported as mean values of mRNA abundance, after normalization with *Cry2* gene. *Error bars* represent SE. Main significances on the text. B-type plant cold treatment described in “Materials and methods”

During the B-type cold treatment inducing artificial acclimation in Canino plants (Fig. 1c), *OeOSM* transcripts sharply increased in its drupes already at day 7 (*P* < 0.0001 in comparison with days 0–2), and did not change further (Fig. 3a). Conversely, *OeOSM* transcripts widely fluctuated in Moraiolo drupes, with significant (*P* < 0.0001) increases at days 2–7 in comparison with day 0, but with a strong decrease, up to day 0 value, at day 14 (Fig. 3a).

To verify whether *OeOSM* transcripts changed in leaves that had experienced long periods at low temperatures under open-air, an experiment was carried out at WAF26 using the Ca^2+^-ionophore A23187, because it causes [Ca^2+^]_cyt_-transients similarly to cold-shocks in the absence of acclimation (D’Angeli et al. 2003). *OeOSM* levels were similar in Canino, and always significantly (*P* < 0.0001) higher than in Moraiolo (Fig. 3b). In the latter, *OeOSM* expression was calcium-modulated, with progressive rises (*P* < 0.05 increases at 48 h in comparison with 0 h, *P* < 0.01 at 72 h in comparison with 48 h, Fig. 3b).

### *OeFAD8* transcript levels changed with cold differently in the presence and absence of cold-acclimation, as C18:3-lipids

The content of the α-linolenic acid (C18:3) in the PL, FFA, and TAG fractions was determined in epi-mesocarp and leaves at WAF19, i.e., when differences in cold-acclimation had appeared in Canino and Moraiolo plants grown under open-air. The total levels of the unsaturated-FA were conspicuously higher in the cold-acclimated genotype than in the sensitive one, independently of the organ, being more than threefold higher in the drupes and more than sixfold in the leaves in the former genotype in comparison with the latter (Fig. S4). Moreover, consistent differences in the total α-linolenic acid content were observed between the two organs in Canino, with the leaves exhibiting a content about fourfold higher than the drupes, whereas only slight differences were observed in Moraiolo (i.e., a 1.5-fold higher content in the leaves compared with the drupes, Fig. S4). Moreover, the percentage distribution of C18:3 in the fractions of each organ changed significantly between the genotypes, with the compound mainly present in Canino in the TAGs of the drupe and PLs of the leaf, and in Moraiolo in the PLs of the drupe and FFAs of the leaf (Table 3).

**Table 3:** Mean percentage distribution (±SE) of C18:3 in polar lipid (PL), free FA (FFA), and triacylglycerol (TAG) fractions in drupes (epi-mesocarp) and leaves of Canino and Moraiolo at WAF19

WAF 19	Drupe Mean % (±SE)	Leaf Mean % (±SE)
Canino		
C18:3(n-3) PL	25.50 ± 0.36^a^	59.26 ± 0.6^a^
C18:3(n-3) FFA	34.47 ± 0.49^b^	25.27 ± 0.26^b^
C18:3(n-3) TAG	40.03 ± 0.57^c^	15.47 ± 0.16^c^
Moraiolo		
C18:3(n-3) PL	51.96 ± 0.85^d^	39.57 ± 0.57^d^
C18:3(n-3) FFA	38.86 ± 0.64^e^	49.88 ± 0.71^e^
C18:3(n-3) TAG	9.18 ± 0.12^f^	10.55 ± 0.15^f^

Comparisons within values of the same column. Different letters show *P* < 0.0001 differences, except for the FFA fractions between Canino and Moraiolo drupes, and the FFA and TAG fractions within Canino drupes (*P* < 0.001 in both cases)

The presence of the oxylipin 13S-hydroxy-9Z,11E,15Z-octadecatrienoic acid (13-HoTre), a C18:3 oxidation-product, was also evaluated, because it is a stress-induced compound and a possible cutin component (Montillet et al. 2005; Blée et al. 2012). In Canino and Moraiolo, it was present in the drupes at not significantly different micromolar levels, i.e., 0.37 (±0.03) and 0.52 (±0.18), whereas at highly significantly (*P* < 0.0001) different levels in the leaves [2.24 (±0.2) and 25.75 (±0.33), respectively].

**Fig. 4:**
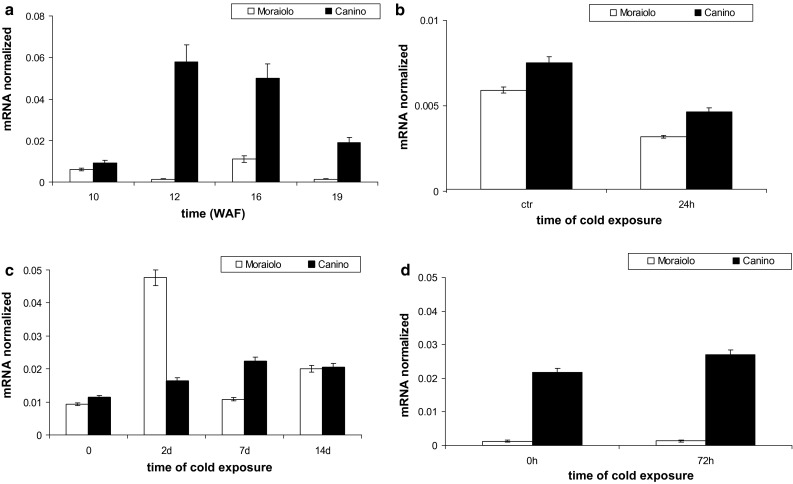
Expression profiles by qPCR of *OeFAD8* transcripts from Moraiolo (*white columns*) and Canino (*black columns*) drupes from WAF10 to WAF19 of plant growth under open-air (**a**), after plant exposure to A_2_-cold treatment at WAF10 in comparison with drupes of same-aged plants maintained at the open-air temperature (25 °C) [control (ctr)] (**b**), during plant exposure to B-type cold treatment starting from WAF10 (**c**), and after plant exposure to A_3_-type cold treatment at WAF19 (**d**). Data reported as mean values of mRNA abundance, after normalization with *Cry2* gene. *Error bars* represent SE. Main significances on the text. A_2_-, B-, and A_3_-type plant cold treatments described in “Materials and methods”

*OeFAD8* was investigated as promising candidate to induce the observed changes in the C18:3 compounds related to acclimation (see “Introduction”). *OeFAD8* expression was low in the drupes at WAF10, without significant differences between genotypes, but with further drupe growth its levels became higher in Canino than in Moraiolo (*P* < 0.0001 differences at WAF12 and WAF16, *P* < 0.05 difference at WAF19, in comparison with WAF10, Fig. 4a).

When the plants were exposed to A_2_-type cold-treatment at WAF10, i.e. at the time, and conditions, not causing cold-acclimation (Fig. S2b), a significant (*P* < 0.01) reduction in *OeFAD8* transcripts occurred in the drupes of both genotypes (Fig. 4b).

When artificial acclimation was induced at WAF10 in Canino and not Moraiolo by the B-type cold treatment (Fig. 1c), in the drupes of the former, after a small, but significant (*P* < 0.05), increase in *OeFAD8* levels at day 2 in comparison with day 0, there was a *P* < 0.0001 rise at day 7, but no further significant variation (Fig. 4c). By contrast, transcript levels transiently changed in Moraiolo drupes, with a highly significant (*P* < 0.0001) increase at day 2, a strong decrease at day 7 (up to day 0 value), and a new increase (*P* < 0.01) at the treatment-end (Fig. 4c). When the naturally (open air)-cold-acclimated WAF19-plants of Canino, and the same-aged, but cold-sensitive, plants of Moraiolo, were exposed to an A_3_-cold treatment, *OeFAD8* levels were very low in Moraiolo, and many-fold higher (*P* < 0.01 differences with Moraiolo), and stable, in Canino (Fig. 4d).

**Fig. 5:**
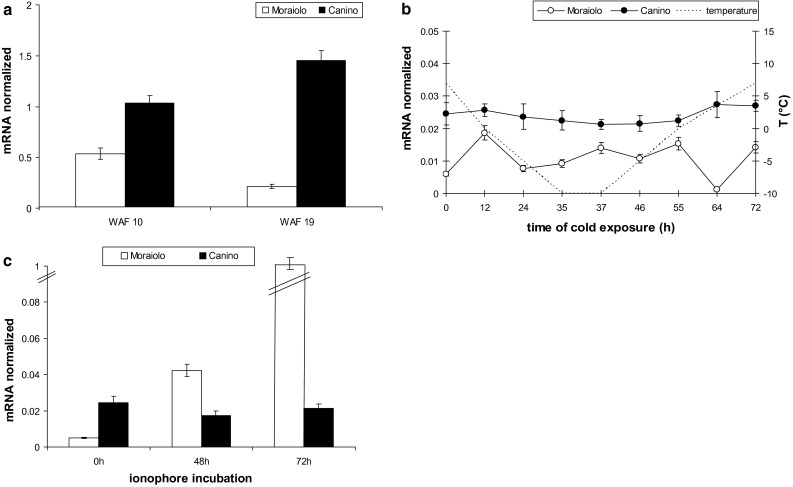
Expression profiles by qPCR of *OeFAD*8 transcripts in the leaves of Canino (*solid symbols*), and Moraiolo (*empty symbols*) at WAF10- and WAF19-plant growth under open-air (**a**), at WAF26 during a C-type cold-exposure of the plants (**b**), and at WAF26 during a 72 h Ca^2+^-ionophore A23187 treatment to the leaves (**c**). Data reported as mean values of mRNA abundance, after normalization with *Cry2* gene. *Error bars* represent SE. Main significances on the text. C-type plant cold treatment described in “Materials and methods”

In both genotypes grown under open-air from WAF10 to WAF19, *OeFAD8* levels in the leaves were higher than in the drupes, but, at WAF19, they were significantly (*P* < 0.01) increased in Canino, and decreased in Moraiolo, as in the drupes (Figs. 4a, 5a). Moreover, the expression at WAF19 of *OeFAD3* and *OeFAD7*, other *ω3*-*FAD* genes, was detected only in traces, excluding any possibility of their cooperation with *OeFAD8*, and the activity of its protein, in producing the C18:3 levels necessary for natural acclimation. When, at WAF26, the plants were exposed to a C-type cold treatment, *OeFAD8* mRNA remained unchanged in Canino leaves, whereas fluctuated in Moraiolo (*P* < 0.001 increases at 12, 37, 55, and 72 h in comparison with 0 h). However, the gene transcripts were highly lowered in comparison with WAF19 (Fig. 5a, b).

The application of the Ca^2+^-ionophore to the leaves at WAF26 showed that *OeFAD8* expression was calcium-modulated in Moraiolo, with strong rises in expression (*P* < 0.0001 increases at 48 and 72 h in comparison with 0 h), whereas it did not change significantly in Canino (Fig. 5c). The possibility that *OeFAD7* and *OeFAD3* might support *OeFAD8* in sustaining leaf acclimation was investigated. However, no relationship of the two genes with cold acclimation was found, because *OeFAD3* expression continued to be in traces, and *OeFAD7* highly fluctuated also in Canino, and even when the plants experienced a C-type cold treatment (Fig. S5).

### A LIP TF is present in olive-tree genome

**Fig. 6:**
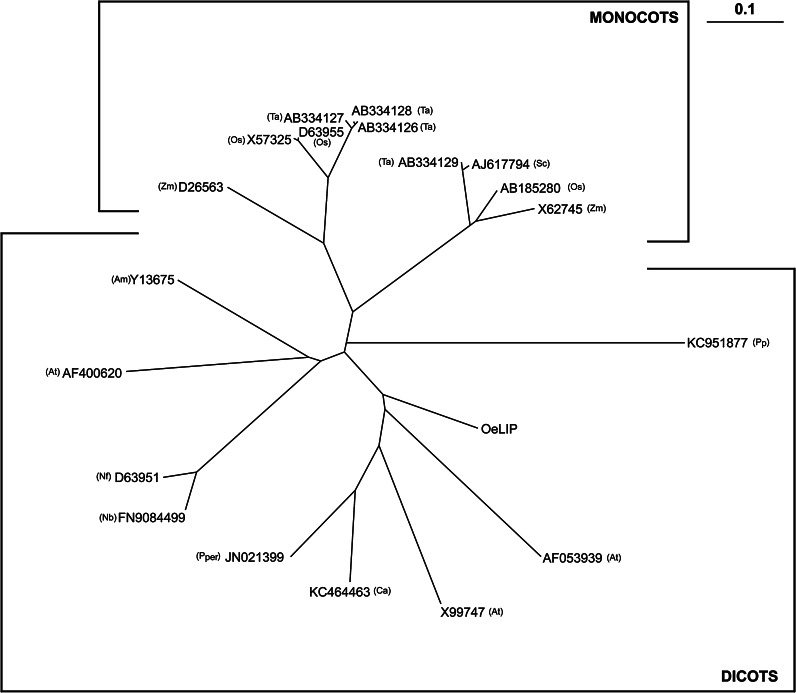
Phylogenetic radial tree of amino acid successions of OeLIP and other 19 dicot and monocot b-ZIP sequences registered in GenBank. Accession no. X57325 = Rice *lip19* mRNA for basic/leucine zipper protein; accession no. AB334128 = *Triticum aestivum* *Wlip19d* mRNA for basic region/leucine zipper protein, complete cds; accession no. AB334126 = *Triticum aestivum* *Wlip19b* mRNA for basic region/leucine zipper protein, complete cds; accession no. AB334127 = *Triticum aestivum* *Wlip19a* mRNA for basic region/leucine zipper protein, complete cds; accession no. D63955 = *Oryza sativa* *glip19* gene, complete cds; accession no. FN908499 = *Nicotiana benthamiana* partial mRNA for bZIP transcription factor (*lip* gene); accession no. KC464463 = *Cicer arietinum* bZip (bZIP) mRNA, complete cds; accession no. KC951877 = *Pyrus pyrifolia* bZIP protein (bZIP) mRNA, complete cds; accession no. JN021399 = *Prunus persica* bZIP transcription factor mRNA, complete cds; accession no. AB185280 = *Oryza sativa* Japonica Group *OsOBF1* mRNA for bZIP protein, complete cds; accession no. AB334129 = *Triticum aestivum* *TaOBF1a* mRNA for basic region/leucine zipper protein, complete cds; accession no. AJ617794 = *Secale cereale* mRNA for ocs-element binding factor 1 (*obf1* gene); accession no. D26563 = *Zea mays* mRNA for *mLIP15* (DNA-binding factor), complete cds; accession no. X62745 = *Z. mays* *OBF1* mRNA for ocs-element binding factor 1; accession no. AF053939 = *Arabidopsis thaliana* transcription factor *GBF5* (*GBF5*) mRNA, complete cds; accession no. X99747 = *A. thaliana* *B2* gene; basic domain/leucine zipper transcription factor; bZIP transcription factor; accession no. D63951 = *Nicotiana tabacum* mRNA for *TBZ17*, complete cds; accession no. Y13675 = *Antirrhinum majus* mRNA for bZIP DNA-binding protein, 1095 bp; accession no. AF400620 = *Arabidopsis thaliana* transcription factor-like protein *bZIP53* mRNA, complete cds). For each accession, the *letters* in *parentheses* identify the species

The presence of a TF homologous to cold-induced *b*-*Zip LIP* genes of other species was investigated. Total cDNA, synthesized starting from RNA samples of Canino, was used as template for PCR amplifications of partial regions of *OeLIP*, as described in “Materials and methods”. Amplicons were sequenced and *OeLIP* nucleotide succession (Table 4) loaded in GenBank (KR360744). The amino acidic sequence of *OeLIP* was also aligned and compared with nineteen well-documented b-ZIP proteins (including LIP proteins) registered in GenBank. Multiple sequence alignment clearly indicated that OeLIP presented the typical features of b-ZIP proteins: the basic domain (underlined sequence, rich in basic amino acids and able to bind the target DNA regions), and the leucine zipper (evidenced by arrows, a series of repeats of leucines or other hydrophobic residues at every seventh position that are responsible for the dimerization of b-ZIP TFs) (Fig. S5). The similarity matrix produced by the comparisons of the b-ZIP protein successions (Table S1) revealed that OeLIP showed a percentage identity of its sequence with the other b-ZIP proteins ranging between 63.37 % (when related with the accession no. JN021399; 67 identical aa and 79 similar aa) and 30.39 % (in correlation with the accession no. KC951877; 34 identical aa and 52 similar aa). According to this matrix, a phylogenetic radial tree was also constructed; OeLIP branch was perfectly inserted in the genetic structure among the dicot b-ZIP proteins (Fig. 6).

**Table 4:** The partial nucleotide sequence of *OeLIP* sequenced starting from Canino genotype

*OeLIP* (ID: KR360744)	Nucleotide sequence
	AGCAGCCGCCTGAAACGCCCGAGCGCGCCGTGGCGCCGCCGCCTGCGCCAGGTGGGCAGCGGCGGCGCGATGGATGAACGCAAACGCAAAATGCTGAGCAACCGCGAAAGCGCGCGCCGCAGCCGCATGCGCAAACAGAAACGCCTGGATGAACTGATGGCGCAGGTGGCGCAGCTGAAAAAAGAAAACCGCCAGATTCTGGCGAGCGTGAACGAAGTGACCCAGCTGTATCTGAACATTGAAGCGGAAAACAACGTGCTGCGCGCGCAGGCGGAAGAACTGAGCGATCGCCTGCAGAGCCTGAAC

### *OeLIP* transcript levels change with cold in drupes and leaves, but differently in the presence and absence of acclimation, and with a trend similar to *OeFAD8*

**Fig. 7:**
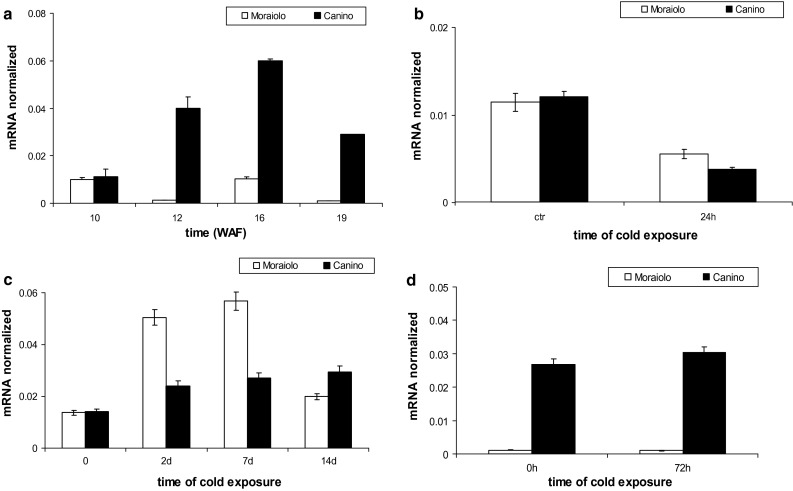
Expression profiles by qPCR of *OeLIP* transcripts in the drupes of Moraiolo (*white columns*), and Canino (*black columns*) from WAF10 to WAF19 of plant growth under open-air (**a**), after plant exposure to A_2_-cold treatment at WAF10 in comparison with drupes of same-aged plants maintained at the open-air temperature (25 °C) [control (ctr)] (**b**), during plant exposure to B-type cold treatment starting from WAF10 (**c**), and after plant exposure to A_3_-type cold treatment at WAF19 (**d**). Data reported as mean values of mRNA abundance, after normalization with *Cry2* gene. *Error bars* represent SE. Main significances on the text. A_2_-, B-, and A_3_-type plant cold treatments described in “Materials and methods”

*OeLIP* expression at WAF10 was similar in Moraiolo and Canino drupes, but further, fluctuated in the former reaching very low levels at WAF19. Conversely, in the latter genotype significant increases (*P* < 0.0001) occurred at WAF12/16, and were followed, at WAF19, by a slight decrease in level, which however, remained higher than WAF10 (*P* < 0.001) (Fig. 7a).

When WAF10-plants were exposed to an A_2_-type cold-treatment, a *P* < 0.01 reduction in *OeLIP* transcripts occurred in the drupes of both genotypes in comparison with the control plants (Fig. 7b). A B-type cold treatment applied to the plants starting from WAF10 caused a *P* < 0.001 increase in *OeLIP* transcripts in Canino drupes at day 2, and constant values after (Fig. 7c). Conversely, highly significant (*P* < 0.0001) *OeLIP* levels were reached in Moraiolo at days 2–7, but were followed by a strong (*P* < 0.0001) decrease (day 14) (Fig. 7c). When the plants were exposed to an A_3_-type cold treatment at WAF19, *OeLIP* levels were quite negligible in Moraiolo, and many-fold higher, and constant, in Canino (Fig. 7d), similarly to *OeFAD8* (Fig. 4d).

**Fig. 8:**
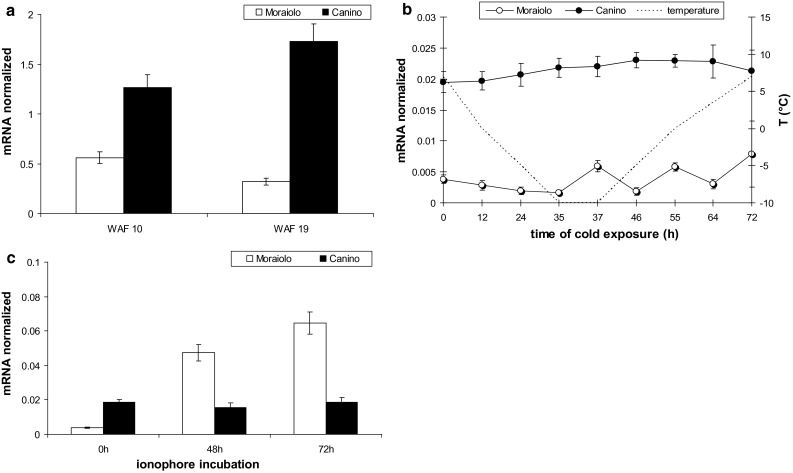
Expression profiles by qPCR of *OeLIP* transcripts in the leaves of Moraiolo (*white columns*/*empty symbols*) and Canino (*black columns*/*solid symbols*), at WAF10 and WAF19 under open air (**a**), at WAF26 during a C-type cold-exposure of the plants (**b**), and at WAF26 during a 72 h Ca^2+^-ionophore A23187 treatment to the leaves (**c**). Data reported as mean values of mRNA abundance, after normalization with *Cry2* gene. *Error bars* represent SE. Main significances on the text. C-type plant cold treatment described in “Materials and methods”

During WAF10 to WAF19 interval, *OeLIP* levels in the leaves of both genotypes growing under open-air were more abundant than in the drupes, and in Canino, in particular (Fig. 8a). Moreover, Canino *OeLIP* abundance increased significantly (*P* < 0.01) at WAF19 in comparison with WAF10, whereas decreased (*P* < 0.01) in Moraiolo (Fig. 8a), as observed for *OeFAD8* (Fig. 5a).

At WAF26, *OeLIP* transcripts in the leaves of both genotypes were many-fold lower than at WAF19 (Fig. 8a, b). However, when the plants were exposed to a C-type cold treatment, *OeLIP* mRNA remained constant in Canino, whereas fluctuated in Moraiolo (Fig. 8b). The ionophore application to the leaves showed that *OeLIP* expression was highly enhanced by calcium-influx in Moraiolo, with progressive rises, i.e., at 48 h (*P* < 0.001 difference with 0 h), and 72 h (*P* < 0.0001 difference with 0 h), whereas did not change significantly in Canino (Fig. 8c), with a response similar to that of *OeFAD8* (Fig. 5c).

## Discussion

### Cold-acclimation of olive tree leaves and drupes needs enhanced cell wall cutinisation, sustained by OeOSM and OeFAD8 activities

Cuticle is a hydrophobic barrier formed in response to various stresses, with water deficit triggering an increase in cuticular thickness as an adaptation response to both heat- (summer) and cold- (winter) drought (Borisjuk et al. 2014). During the summer, olive tree, like other Mediterranean xerophytes, may be subjected to high air temperatures, high vapour pressure deficits, and limited water availability, but differences in cuticle thickening exist among its genotypes in response to this heat-caused drought-stress (Bacelar et al. 2004). Present data show that a positive relationship between increased cuticle thickening and cold acclimation exists in olive tree, because the aerial organs (leaves and drupes) of the cold-acclimated Canino, and Frantoio, showed a thicker cuticle than those of the cold-sensitive Moraiolo. This increased cutinisation may be interpreted as an adaptation of specific genotypes to strongly limit dehydration during winter by enhancing the resistance to water loss by the cuticle formed during summer. Cutin, i.e., the cuticle polymer matrix, consists of monomers assembled intracellularly, with larger oligomers (cutinsomes, Heredia-Guerrero et al. 2008) requiring exocytosis of OBs outside the plasma membrane (Pollard et al. 2008). Exocytosis of OBs occurs in olive tree seeds (D’Angeli et al. 2013), and here is shown that it also occurs in the leaf epidermis and in fruit epicarp, with OB-flux also interesting innermost cell layers, in accordance with observations in other plants (Martin and Rose 2014). Lipotubuloids, participating with cutinsomes in cuticle formation through the activity of lipid-transfer proteins, have been described in *Ornithogalum umbellatum* fruit epidermis (Kwiatkowska et al. 2014). The micro-channel-like striations presently observed in the cutinized outer-cell-walls of olive-tree leaf epidermis/epicarp resemble these structures, and show OeOSM immuno-localization signal. Osmotin acts in cryoprotection and dehydration tolerance in numerous plants, and is involved in the extracellular export of cutinsomes in olive tree seeds in response to cold (D’Angeli et al. 2013). Because its presence increased in Canino leaves/drupes at acclimation, a role for the protein, related to acclimation acquisition, by increasing the apoplastic translocation of cutinsomes is plausible. The maintenance of a high *OeOSM* transcription in the following WAFs in Canino, and not in Moraiolo, suggests that an elevated OeOSM activity might contribute to maintain high dehydration tolerance during acclimation persistence.

In the OBs of olive tree drupe, the TAGs are the main storage lipids (Salas et al. 2000). They too might participate to cutinisation, being involved in the formation of hydroxyl-FAs, as occurs in *Arabidopsis* (Rani et al. 2010). Present results show that C18:3 levels were high in the TAGs of Canino drupes at WAF19. It is possible that they were needed to sustain the increasing epicarp cutinisation of the following WAFs, necessary to maintain cold-hardiness in these fruits which remain on the tree for a long time. Conversely, in Moraiolo drupes, fated to fall rapidly, C18:3 was detected at low levels, but preferentially in the PLs. It is possible that, under open air, a short-term response to the temperature-lowering at this WAF (still above 0 °C) was activated by these drupes, and involve an enrichment in C18:3-PLs, as in the cold-short-term response of *Arabidopsis* (Burgos et al. 2011).

Canino showed the highest levels of C18:3 in the leaves, and in the C18:3-PLs, in particular. This suggests that the leaves, unable to further increase cutinisation, differently from the drupes, maintained cold adaptation by enhancing the fluidity of their membranes. In accordance, the electrolyte leakage test at WAF19, i.e., the WAF of acclimation under open-air, showed that the leaves of Canino exhibited a significantly reduced membrane damage in comparison with their drupes, and the leaves and drupes of Moraiolo. Similar changes in the membrane-lipidome in response to cold, causing enhanced cold resistance, have been reported also for *Arabidopsis* leaves (Routaboul et al. 2000). Moreover, in cowpea, a high C18:3-unsaturation characterizes the leaf membranes of a drought-tolerant cultivar, but not of a sensitive one (Torres-Franklin et al. 2009). This suggests, together with present data, a relationship between cold and drought long-term adaptation involving similar changes in the C18:3 of leaf membranes.

In the cold-sensitive leaves of Moraiolo free C18:3 increased significantly in comparison with the other fractions. It is possible that it was necessary for producing oxylipins via reactive oxygen species (ROS), known to be triggered by the cold stress (Prasad et al. 1994). In accordance, increased levels of the 13-HoTre oxylipin characterized these leaves. Moreover, Moraiolo leaves also showed reduced levels of *OeOSM* in comparison with the other genotype. These reduced levels might be involved in the ROS enhancement, and the reduced cold-defence of Moraiolo, because osmotin is known, among the other functions, to help in the accumulation of the osmolyte proline, which quenches ROS and free radicals (Kumar et al. 2015, and references therein).

In all cases, however, present data show that many-fold higher levels of C18:3 (all fractions) were present in Canino in comparison with Moraiolo, when acclimation occurred in the former and not in the latter (WAF19). Because the expression of the *OeFAD* genes, responsible with *OeFAD8*, of linoleic acid desaturation leading to C18:3 production, i.e., *OeFAD3* and *OeFAD7,* was irrelevant at this time in both genotypes, and only *OeFAD7* was present later, but with a genotype-independent fluctuating expression, results show that cold acclimation specifically couples with *OeFAD8* expression/OeFAD8 activity in olive tree. In rice the same direct relationship between the low-temperature-dependent C18:3-changes and the transcriptional regulation of *OsFAD* has been suggested (Gopalakrishnan Nari et al. 2009).

Results showed that *OeFAD8* exhibited lower levels during advanced acclimation in comparison with early acclimation, suggesting a lower necessity of OeFAD8-mediated C18:3 production to maintain acclimation during advanced winter. Conversely, in Moraiolo, *OeFAD8* levels continued to be low and fluctuating, but increased in full-winter leaves under the calcium-ionophore-treatment. Because Ca^2+^-ionophores induce [Ca^2+^]_cyt_-transients similarly to cold-shocks in the absence of acclimation (D’Angeli et al. 2003, and references therein), it is possible that the gene was activated by calcium-transients for allowing a certain production of C18:3 as an attempt of inducing, at least, a short-term cold protection in the leaves of this genotype.

### OeLIP is involved in olive tree cold acclimation

The LIP19 subfamily includes members that might be involved in cold acclimation (Kobayashi et al. 2008). These bZIP-TFs bind DNA sequences containing the ACGT motif, while the sequence flanking this core-region is quite variable according to nature of the target gene and the specificity of the factor involved in the regulation (Izawa et al. 1993; Kusano et al. 1995). Thus, members of the bZIP family have different (although often overlapping) binding site preferences, with the main group binding to the CACGTG (G-box) motif. However, there are bZIP-TFs, belonging to the subfamily, including LIP19 from rice and mlip15 from maize, that bind to a hybrid C-box/G-box motif (Martínez-García et al. 1998). Our results show that an *OeLIP,* exhibiting the typical features of b-ZIP proteins, and perfectly inserted in the genetic structure among the dicot LIP19 proteins, is present in olive tree genome and its expression is positively related with cold acclimation acquisition and maintenance. However, except the ACGT region, other peculiar nucleotide sequences (or the flanking regions of the ACGT motif) present on OeLIP-target genes, and that should be involved in the interactions with this TF during cold acclimation, remain to be determined. In *Arabidopsis,* the activation of *Rab18*, a *Cor* (cold-responsive)/*Lea* (Late embryogenesis abundant) gene, coding a dehydrin, is strongly associated to low-temperature stimuli and cold acclimation (Mäntylä et al. 1995). In olive tree, an *OeRab18* gene has been identified in the seed, with its expression positively related to cold- and desiccation-tolerance (D’Angeli et al. 2013). For this gene, the possibility that the LIP protein may act as TF is high, and sustained by our preliminary results, which need confirmation by the completion of *OeRab18* sequencing. However, in accordance with our hypothesis, an interaction of Wlip19 with wheat *Cor/Lea* gene promoters, including *WRab18,* has been demonstrated in common and winter wheat (Kobayashi et al. 2008; Sun et al. 2009).

Moreover, we presently show that *OeLIP* expression parallels that of *OeFAD8* during acclimation. Also in maize, the expression profiles of *ZmFAD8* and *mlip15* (a *LIP19* gene) were similar under prolonged cold-exposure (Berberich et al. 1998). An up-regulation by low temperatures was also reported for *rdlip* in radish (Ito et al. 1999), and low-temperature-induced levels of a *lip19* were maintained stable in rice (Shimizu et al. 2005).

Again, as for *OeFAD8, OeLIP* levels increased in the full winter leaves of Moraiolo under the calcium-ionophore-treatment, sustaining that, in the absence of acclimation, both genes were activated by cytosolic calcium transients to alleviate prolonged cellular cold stress.

In *Arabidopsis*, the plasma-membrane-bound NTL6 TF integrates cold signals into plant defense responses, because after cold activation, its transcriptionally active form (NAC) is cleaved and goes to the nucleus to induce *PR*-*5* genes (Seo et al. 2010). An *OeNTL6* gene is induced by cold in olive tree seed, with its NAC inducing *OeOSM,* and OeOSM acting in lipid-trafficking for endosperm cutinisation (D’Angeli et al. 2013). An effect of NTL6 on early activating bZIP proteins to alleviate stress has been recently shown (Yang et al. 2014), and our preliminary results sustain that OeNTL6 activates not only *OeOSM*, but also *OeLIP* (Altamura et al. unpublished).

**Fig. 9:**
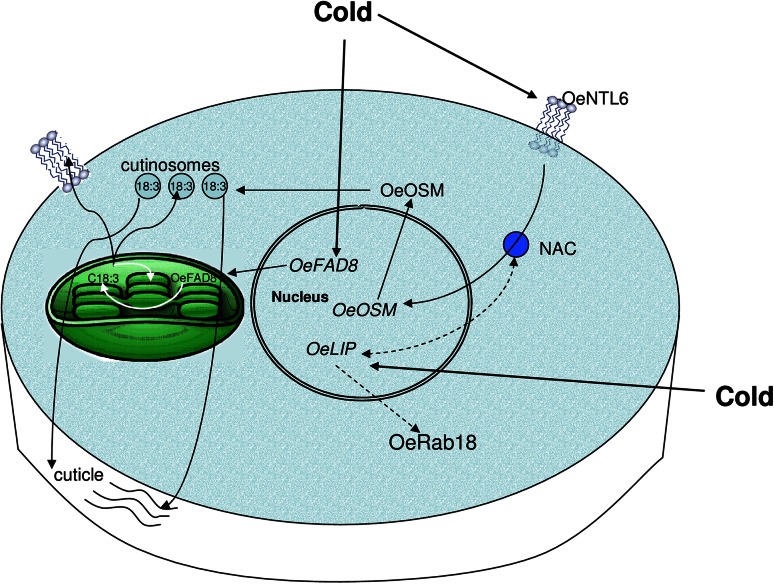
Model of cold-acclimation induction in olive tree drupes and leaves by the orchestrated activities of OeFAD8, OeOSM, and OeLIP. The main cellular mechanisms that come into play are summarized, that is, the cold-induced activation in the nucleus of *OeFAD8* and *OeLIP*, and of *OeOSM* by the NAC-domain coming from the cold-induction of the plasma-membrane OeNTL6, the formation of OeFAD8 in the chloroplast, and the related synthesis of C18:3, the increase of chloroplast- and plasma-membrane fluidity by C18:3-PLs (leaf prevalent mechanism), and the formation of C18:3-enriched cutinsomes, extruded by OeOSM into the cell wall to increase cutinisation (drupe prevalent mechanism). The possible production of OeRab18 by OeLIP activity, and the relationship between *OeLIP* and NAC, are shown by the *dashed lines*

In conclusion, as proposed in Fig. 9, three genes, i.e., *OeFAD8, OeOSM,* and *OeLIP19,* participate to olive tree cold acclimation starting from its induction. They code proteins with different activities, i.e., a FAD, a PR-5, and a TF, respectively, but orchestrated for causing and maintaining cold acclimation, possibly through an increase in dehydration tolerance.

#### *Author contribution statement*

All Authors contributed to the writing of the manuscript and approved it.

## Electronic supplementary material

Below is the link to the electronic supplementary material.

Supplementary material 1 (DOCX 559 kb)

## Abbreviations

FAs: Fatty acids
FADs: Fatty acid desaturases
FFA: Free fatty acid
OBs: Oil bodies
OeOSM: Osmotin
PL: Polar lipid
TAG: Triacylglycerol
TF: Transcription factor
WAFs: Weeks after flowering
