# Supplementary material for: OeFAD8, OeLIP and OeOSM expression and activity in cold-acclimation of Olea europaea, a perennial dicot without winter-dormancy
Source: Planta. 2016 Feb 26;243:1279–96. doi: 10.1007/s00425-016-2490-x (PMC4837226; doi:10.1007/s00425-016-2490-x)

Supplementary Data

Planta

Title:

***OeFAD8, OeLIP* and *OeOSM* expression and activity in cold-acclimation of *Olea europaea*, a perennial dicot without winter-dormancy**

Authors:

**Simone D’Angeli, Maya Matteucci, Laura Fattorini, Angelo Gismondi, Matteo Ludovici, Antonella Canini, Maria Maddalena Altamura**

**Supporting information: 6 figures and 1 table**

**Fig. S1** **a**-**e** Variations in [Ca ^2+^]_cyt_ expressed as pixel intensities (A.U., Arbitrary Units) in Canino and Moraiolo protoplasts of leaf (**a**-**b**) and epi-mesocarp (**c**-**d**) incubated with Calcium-Crimson-AM under a cold-shock of *Δ*T*/Δ*t=10°C/60s from WAF10-plants (**a**,**c**) and WAF19-plants (**b**,**d**), and in Frantoio leaf protoplasts from WAF22-plants (**e**). *n*=80. Protoplasts representative of the average response shown. [5.3(±0.3) and 12.1(±0.8) (**a**), and 11.8(±0.9) and 18.6(±1.1) (**c**) mean rises in Canino and Moraiolo, respectively; 4.9(±0.4) (**b**) and 8.5(±0.8) (**d**) mean rises in Moraiolo]. No rise in Frantoio (**e**). Protoplasts isolated from plants grown under open-air

**Fig. S2 a-c** Variations in [Ca ^2+^]_cyt_ expressed as pixel intensities (A.U., Arbitrary Units), in Canino and Moraiolo protoplasts of epi-mesocarp (**a**,**b**) and leaf (**c**), incubated with Calcium-Crimson-AM under a cold-shock of *Δ*T*/Δ*t=10°C/60s from WAF10-plants that had been exposed either to an **A_1_**-type (**a**) or an **A_2_**-type (**b**) cold treatment, and from WAF26-plants grown under open-air (**c**). *n*=80. Protoplasts representative of the average response shown. [8.9(±0.6) and 5.2(±0.5) (**a**), and 7.6(±0.7) and 3.4(±0.5) (**b**) mean rises in Moraiolo and Canino, respectively; 3.8(±0.4) mean rise in Moraiolo (**c**)]. **A_1_-** and **A_2_**-type plant cold treatments described in Materials and methods

**Fig. S3** Mean percentage of ion (electrolyte) leakage from the cell membranes of Moraiolo (empty symbols) and Canino (solid symbols) leaf and drupe discs excised from WAF10-plants that had been exposed to either **A_3_**-type cold treatment (treat) or **B**-type cold treat, in comparison with WAF10-plants maintained at 25°C (no cold-treat). Error bars represent SE. Significant differences on the text. Ion leakage measurements, and **A_3_-** and **B**-type plant cold treatments described in Materials and methods

**Fig. S4** Comparison of linolenic acid [C18:3(n-3)] content in the lipid fractions [i.e., free fatty acid (FFA), polar lipid (PL), and triacylglycerol (TAG)  fractions] from drupes (epi-mesocarp tissues) and leaves of Canino (solid columns) and Moraiolo (empty columns) at WAF19. Values from gas-chromatography (GC) analysis are expressed as mean areas mg^-1^DW(±SE). The percentage distribution of C18:3 in the three fractions from each organ and the statistical comparison are reported in Table 3

**Fig. S5** Expression levels by qPCR of *OeFAD7* in the leaves of Canino and Moraiolo WAF26 plants exposed to **C**-type cold treatment (see Materials and Methods). Data reported as mean values of RNA abundance, after normalization with *Cry2* (three replicates). Error bars represent SE

**Fig. S6**


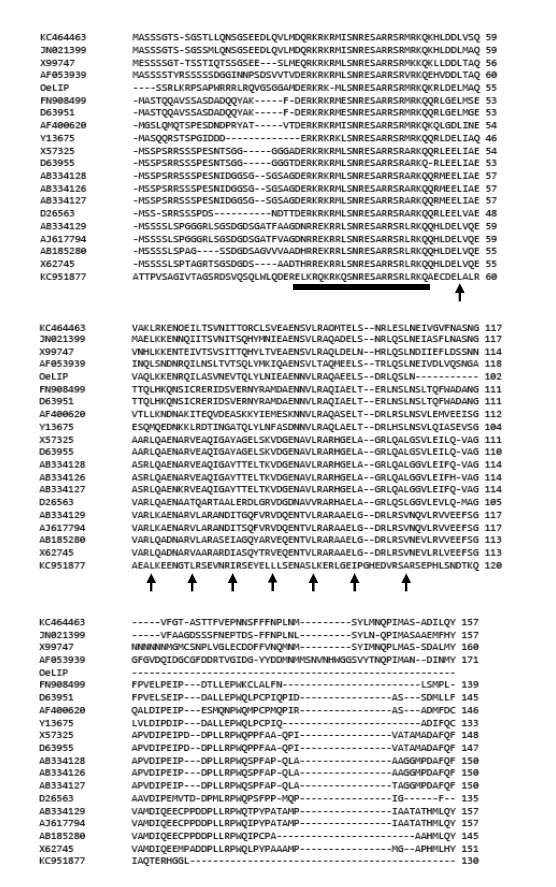


**Fig. S6** Multiple sequence alignment performed using ClustalW2.1 program and loading on the software the protein sequence of OeLIP (GenBank KR360744) and 19 other amino acidic successions relative to b-ZIP proteins registered in GenBank (accession no. X57325 = Rice lip19 mRNA for basic/leucine zipper protein; accession no. AB334128 = *Triticum aestivum* Wlip19d mRNA for basic region/leucine zipper protein, complete cds; accession no. AB334126 = *Triticum aestivum* Wlip19b mRNA for basic region/leucine zipper protein, complete cds; accession no. AB334127 = *Triticum aestivum* Wlip19a mRNA for basic region/leucine zipper protein, complete cds; accession no. D63955 = *Oryza sativa* glip19 gene, complete cds; accession no. FN908499 = *Nicotiana benthamiana* partial mRNA for bZIP transcription factor (lip gene); accession no. KC464463 = *Cicer arietinum* bZip (bZIP) mRNA, complete cds; accession no. KC951877 = *Pyrus pyrifolia* bZIP protein (bZIP) mRNA, complete cds; accession no. JN021399 = *Prunus persica* bZIP transcription factor mRNA, complete cds; accession no. AB185280 = *Oryza sativa* Japonica Group OsOBF1 mRNA for bZIP protein, complete cds; accession no. AB334129 = *Triticum aestivum* TaOBF1a mRNA for basic region/leucine zipper protein, complete cds; accession no. AJ617794 = *Secale cereale* mRNA for ocs-element binding factor 1 (obf1 gene); accession no.D26563 = *Zea mays* mRNA for mLIP15 (DNA-binding factor), complete cds; accession no. X62745 = *Z. mays* OBF1 mRNA for ocs-element binding factor 1; accession no. AF053939 = *Arabidopsis thaliana* transcription factor GBF5 (GBF5) mRNA, complete cds; accession no. X99747 = *A. thaliana* B2 gene; basic domain/leucine zipper transcription factor; bZIP transcription factor; accession no. D63951 = *Nicotiana tabacum* mRNA for TBZ17, complete cds; accession no. Y13675 = *Antirrhinum majus* mRNA for bZIP DNA-binding protein, 1095 bp; accession no. AF400620 = *Arabidopsis thaliana* transcription factor-like protein bZIP53 mRNA, complete cds). The basic conserved domain is underlined with a black bar while the Leucines or the other hydrophobic residues are indicated by arrows

**Table S1** Percentage similarity matrix obtained by the multiple sequence alignment of b-ZIP proteins reported in GenBank and OeLIP aminoacid sequence. Accessions used for the creation of the matrix were reported *in extenso* in the legend of Fig S6


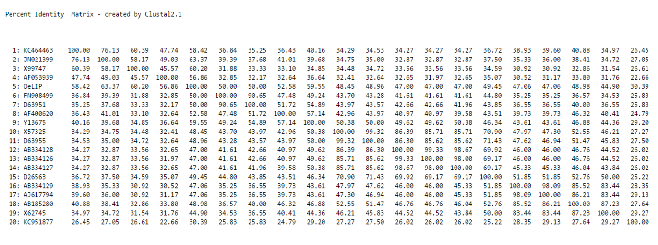

Supplement: Supplementary file 1 — Supplementary material 1 (DOCX 559 kb) [file 425_2016_2490_MOESM1_ESM.docx]
